# Multiple evanescent white dot syndrome following rabies vaccination: a case report

**DOI:** 10.1186/s12886-018-0968-y

**Published:** 2018-12-07

**Authors:** Jia-song Yang, Chun-li Chen, Yu-zhang Hu, Rui Zeng

**Affiliations:** 1 Department of Ophthalmology, Apex Eye Hospital, Henan Province, China; 2 Department of Ophthalmology, Tianjin Medical University Eye Hospital, Tianjin, China; 3 Department of Ophthalmology, Shengli Oilfield Central Hospital, Shandong Province, China; 4 Department of Ophthalmology, Chengdu Aidi Eye Hospital, Chengdu, China

**Keywords:** Rabies vaccine, Multiple evanescent white dot syndrome, Vaccination/adverse events

## Abstract

**Background:**

To report a case of multiple evanescent white dot syndrome (MEWDS) following simultaneous rabies vaccination.

**Case presentation:**

Review of the clinical, laboratory, photographic, optical coherence tomographic (OCT), fundus autofluorescent, angiographic, electrophysiologic, and perimetric records of a patient suffering from MEWDS. Results: A healthy 33-year-old Chinese female suffering from rapidly progressive visual loss of her left eye associated with photopsia and a para-central scotoma, seven days after receiving simultaneous rabies vaccination. Both anterior segments and fundus examination were unremarkable. The findings on OCT, electrophysiology, and perimetry were pathognomonic for MEWDS.

**Conclusions:**

The clinical presentation and the benign course were consistent with the diagnosis of MEWDS. No other events could be identified as a cause, other than the rabies vaccination. This case may suggest an autoimmune basis for MEWDS in predisposed patients.

## Background

In 1984, Jampol described a new chorioretinal disorder named multiple evanescent white dot syndrome (MEWDS) which clinically presents in young, often myopic women with unilateral visual blurring and photopsia [1]. Most patients were young adult women and may be associated with visual field loss and abnormalities on the electroretinographic records [2].

Rabies is a zoonotic disease caused by a virus and affects domestic and wild animals and is spread to people through close contact with infectious material. In unvaccinated humans, rabies is nearly always fatal after neurological symptoms have developed. Rabies is a vaccine-preventable disease. Post-exposure prophylaxis through vaccine and immunoglobulin given soon after exposure to rabies virus is highly effective in preventing rabies [3].

While there were several reports of MEWDS development following several different vaccines administration [4–7], this is the first of MEWDS following rabies vaccination.

## Case presentation

A 33-year-old Chinese female complained of sudden onset of a para-central large scotoma in her left eye seven days after the third intramuscular administration of rabies vaccine (Rabipur, Novartis) according to standard vaccine time schedule (days 0, 3, 7, 14 and 28) for a stray cat scratch. The type of the rabies vaccine is embryonated-egg vaccine. She has not been administered with other vaccines recently and it was the first time she had symptoms after vaccination. The stray cat was not available for observation of rabies symptoms. Otherwise healthy, the patient has no remarkable medical history or underlying pathology. Her best-corrected visual acuity (BCVA) at the time was 20/20 in both eyes with − 9.0 D right eye and − 8.5 D left eye. An ophthalmoscopic examination of the left eye revealed nothing to explain her complaints. During the follow-up, she developed photopsia in the left eye.

**Fig. 1:**
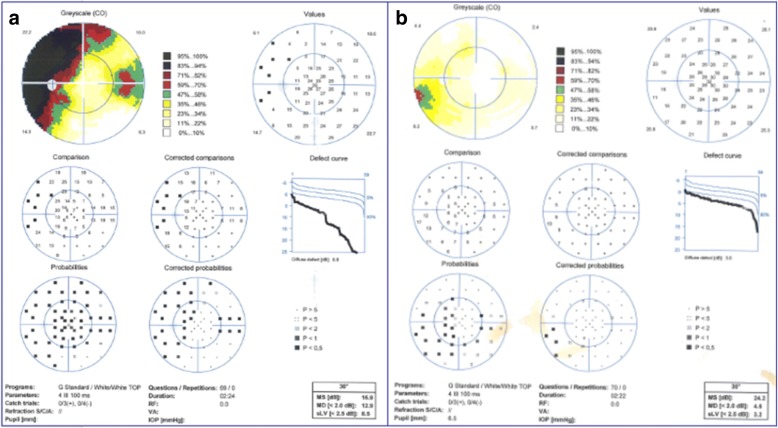
**a**. Octopus visual field showed an enlarged blind spot and decreased sensitivity superiorly and nasally. **b**. 2 months later, the visual field resolved

**Fig. 2:**
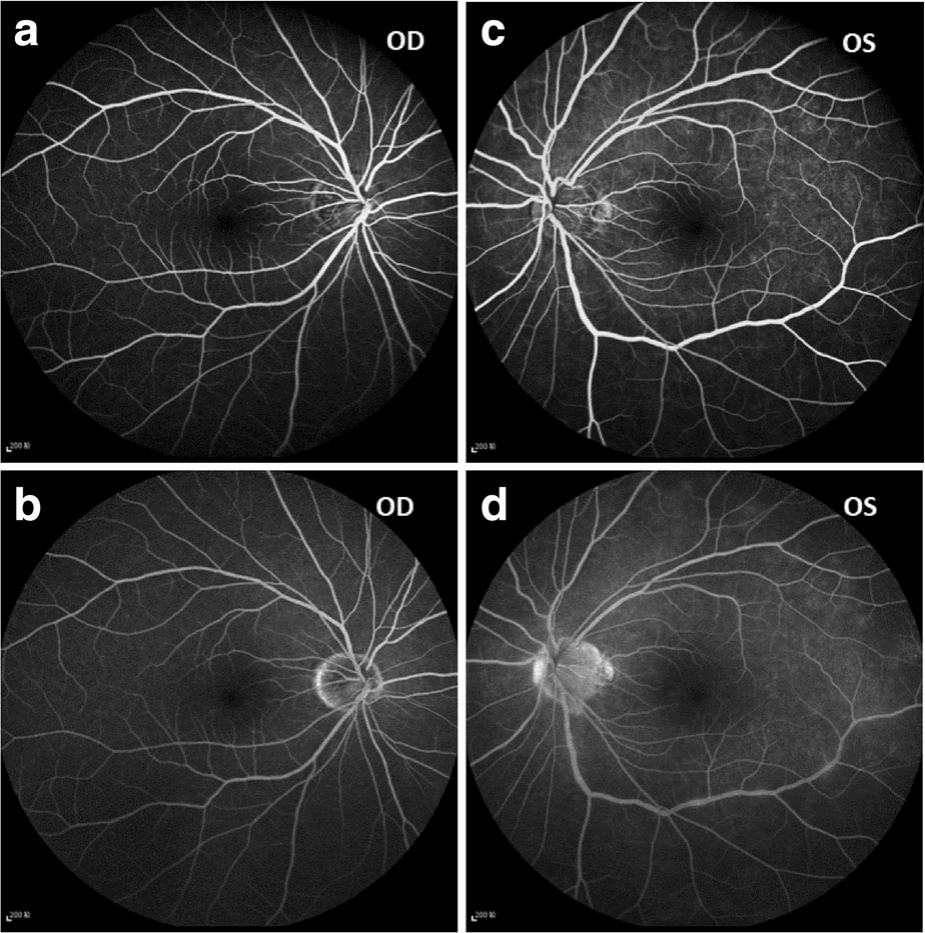
FFA showed right eye the normal appearances in arteriovenous phase (**a**) and late phase (**b**), and early choroidal background hyperfluorescence (**c**) and mild diffuse leakage of fluorescein was noted in the late phase (**d**) of the left eye

**Fig. 3:**
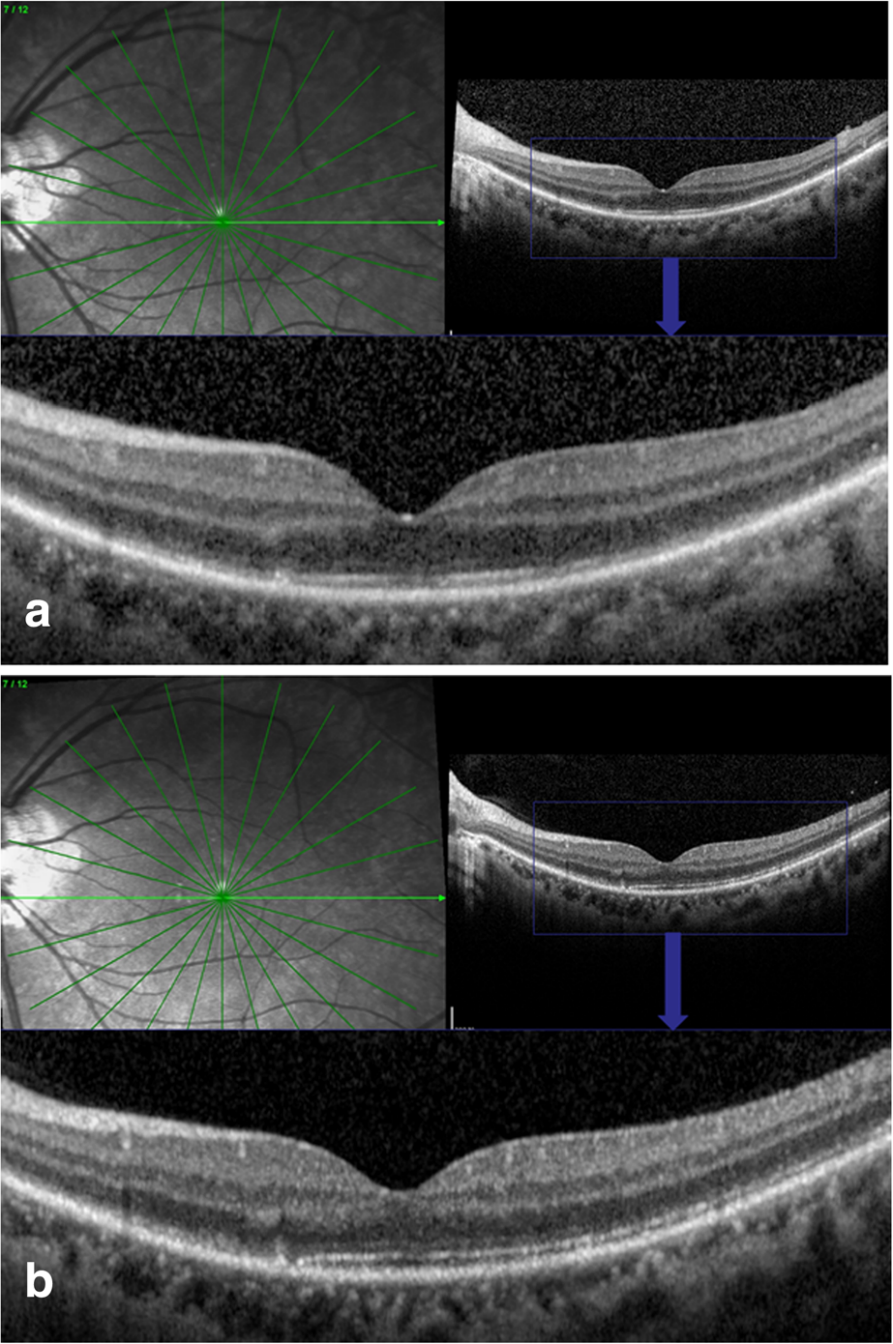
**a**. Optical coherence tomograpy scan showed the interrupted ellipsoid zone of the left eye at the initial visiting time. **b**. The ellipsoid zone partially resolved 2 months later

**Fig. 4:**
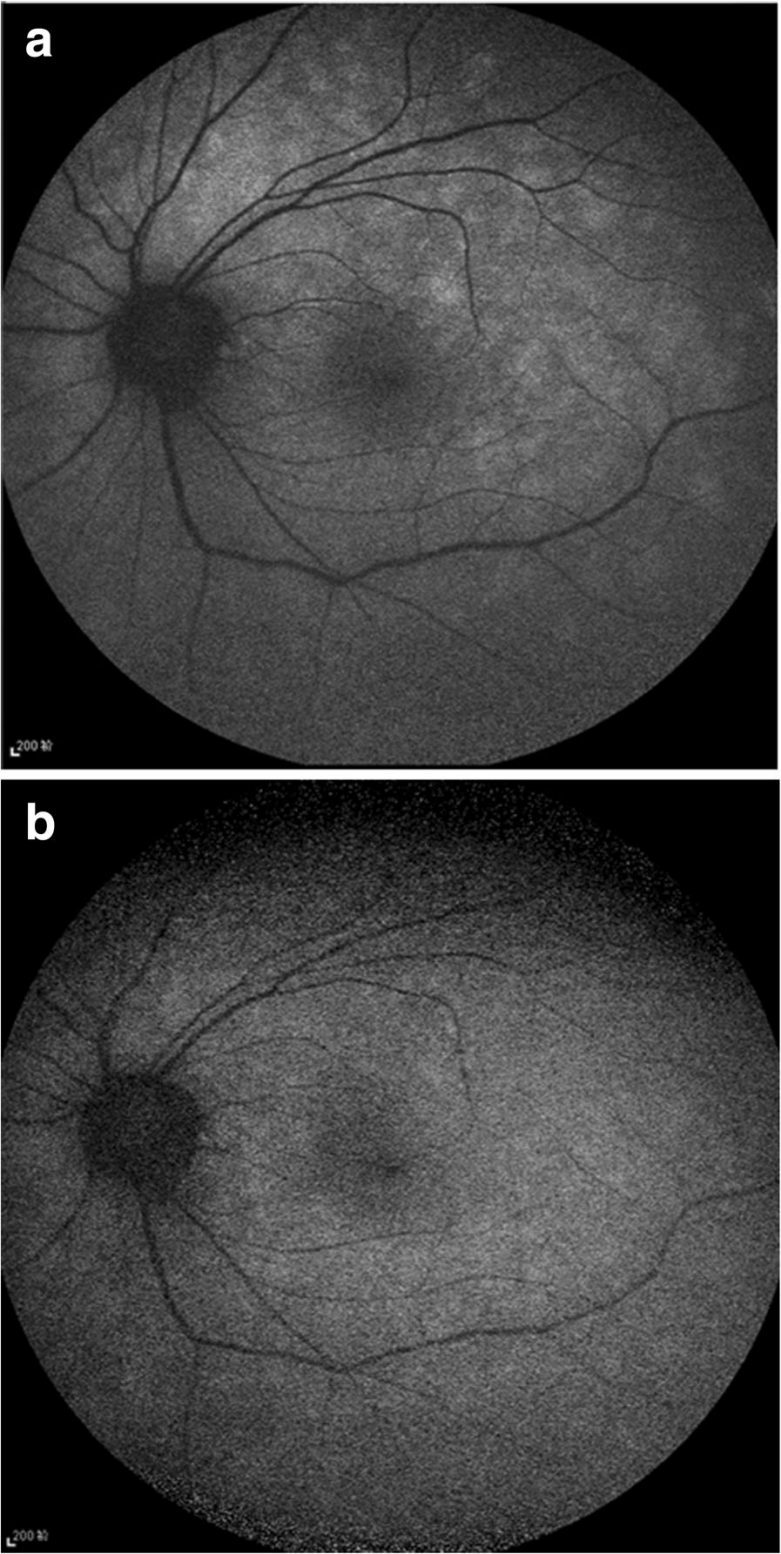
**a**. Fundus AF demonstrated multiple ill-defined spots of markedly increased AF in the posterior pole. **b**. 2 months later, the hyerfluoscent spots decreased on AF

Visual field testing showed an enlarged blind spot and decreased sensitivity superiorly and nasally (Fig. 1a). Fundus fluorescence angiography (FFA) and autofluorescence (AF) revealed hyperfluorescence corresponding to the area of the retina in the region of ellipsoid zone abnormalities. FFA showed right eye the normal appearances in arteriovenous phase (Fig. 2a) and late phase (Fig. 2b), and early choroidal background hyperfluorescence (Fig. 2c) and mild diffuse leakage of fluorescein was noted in the late phase (Fig. 2d) of the left eye. Optical coherence tomography (OCT) demonstrated disruptions in the ellipsoid zone of the posterior retina (Fig. 3a). Fundus AF demonstrated multiple ill-defined spots of markedly increased AF in the posterior pole (Fig. 4a).

**Fig. 5:**
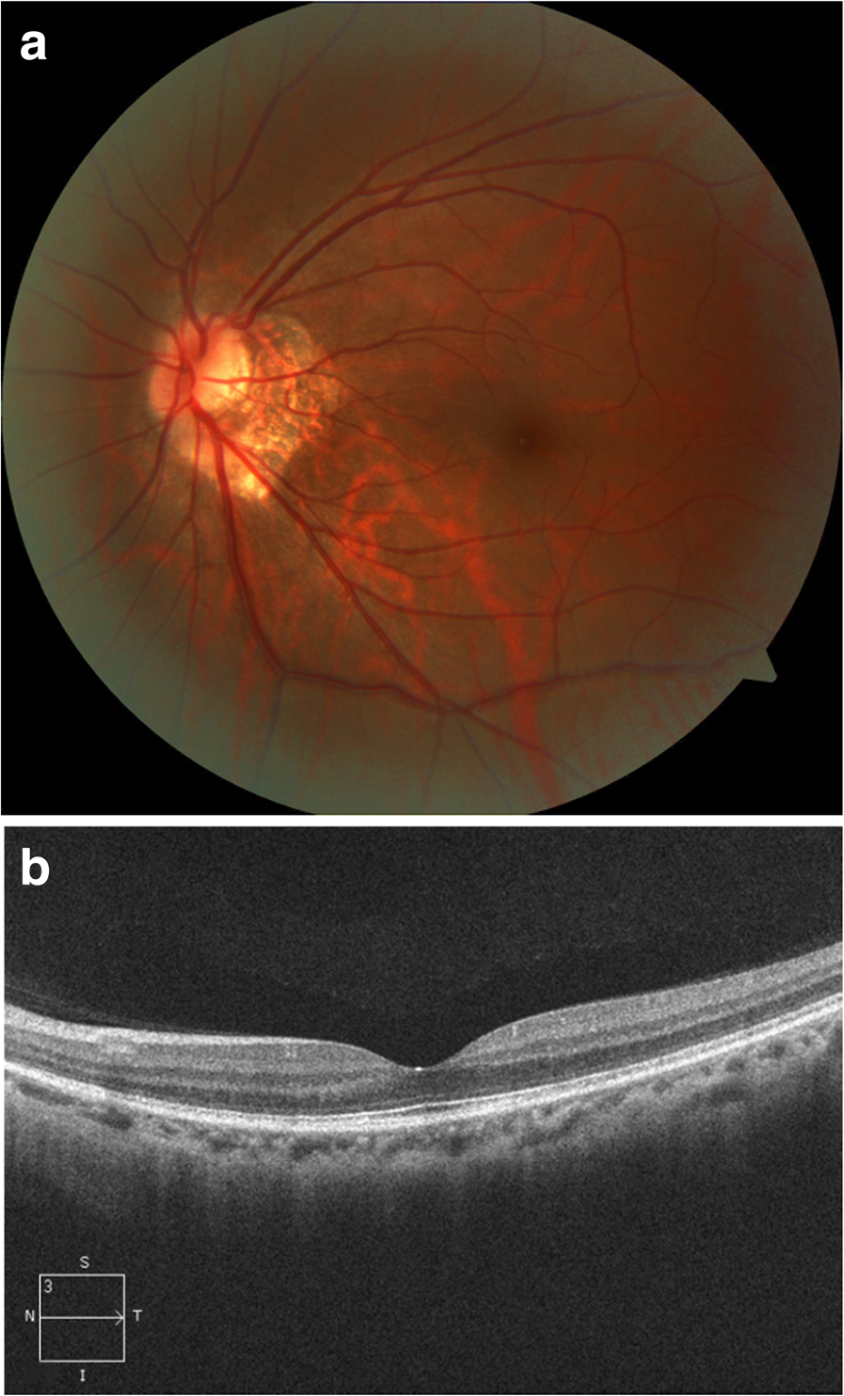
**a** & **b**. Three years later, the fundus was completely restored to normal both on fundus phtograph and OCT

Initial work-up for a complete blood cell count, immunological test, syphilis, human immunodeficiency virus (HIV), and brain computed tomography (CT) were all negative. MEWDS was high in the differential diagnosis. As the patient refused to take oral steroid, she received local steroid injection (retrobulbar injection of triamcinolone acetonide 40 mg). At the follow-up examination 2 months after the initial evaluation, symptoms, OCT (Fig. 3b), Fundus AF (Fig. 4b), and the visual field (Fig. 1b) were partially resolved. Three years later, the patient was examined again, the fundus was completely restored to normal both on fundus photograph and OCT (Fig. 5 a&b) and the BCVA was 20/20 and all symptoms disappeared.

## Discussion

This case, to our knowledge, represents the first reported ophthalmic association following rabies vaccination. Due to the authors’ lack of experience in diagnosing MEWDS, we did not notice the foveal granularity or macular granity during the first visit. As no fundus abnormalities were recognized during the patient initial visit, the fundus data were not preserved. However, there are signs that support the diagnosis. The symptoms and signs of this case as well as the auxiliary examinations are consistent with the characteristic of MEWDS: photopsia, disruption of the ellipsoid zone and the interdigitation layer at the center of the fovea, which is coincidence with what was observed by Cahuzac et al. [8], multifocal hyperautofluorescent lesions at posterior pole and midperiphery, visual field defect corresponding the outer retinal abnormalities. Follow up the entire course of this case revealed complete recovery of the macular and retinal structures and function consistent the natural history of MEWDS.

The differential diagnosis of MEWDS and acute zonal outer retinopathy (AZOOR) is difficult, especially in the early stages of the two conditions. We misdiagnosed the patient as AZOOR at the first consultation. In 2014, Yannuzzi described a specific definition of AZOOR based on multimodal imagings that differ from other white spot syndromes of the posterior fundus [9]. In that retrospective study, Yannuzzi proposed the most characteristic fundus imaging changes of AZOOR: the trizonal pattern changes involving outer retina, retinal pigment epithelium, and choroid, and the demarcating line at the outer retina as the lesion progresses. Our patient’s outer retina was fully recovered, and the visual field defect returned to normal during short-term follow-up. There were also reports of MEWDS combined with AZOOR, but rapid recovery and good prognosis of this case, the report was confirmed as MEWDS rather than AZOOR.

There have been several reports of multiple evanescent white dots syndrome (MEWDS) after receiving the influenza vaccination, human papilloma virus and meningococcus vaccination, hepatitis A vaccination, and yellow fever vaccination [4–7]. Considering the patient’s recent vaccination, the typical symptoms and ancillary tests characteristic of MEWDS, the rabies vaccination may have induced her retinal abnormalities. The etiology of this entity remains uncertain. MEWDS often occurs in patients after a flu-like illness and an autoimmune mediated inflammatory mechanism could explain the onset of MEWDS in our patient.

We still cannot rule out the coincidental association between MEWDS and the rabies vaccination. This is the limitation of a single case report. However, due to the extensive use of rabies vaccines, ophthalmologists need to consider the conditions discussed and related diagnoses when they encounter similar conditions.

## Conclusions

The clinical presentation and the benign course were consistent with the diagnosis of MEWDS. No other events could be identified as a cause, other than the rabies vaccination. This case may suggest an autoimmune basis for MEWDS in predisposed patients.

## Abbreviations

AF: Autofluorescence
AZOOR: Acute zonal occult outer retinopathy
BCVA: Best-corrected visual acuity
CT: Computed tomography
HIV: Human immunodeficiency virus
MEWDS: Multiple evanescent white dot syndrome
OCT: Optical coherence tomography
